# Supplementary material for: Reduced inhibition of hippocampal adult-born granule cells by parvalbumin interneurons after TBI
Source: bioRxiv. 2026 Sep 14:2026.09.08.750063. Preprint. [Version 1] doi: 10.64898/2026.09.08.750063 (PMC13596178; doi:10.64898/2026.09.08.750063)
Supplement: Supplementary file 1 [file NIHPP2026.09.08.750063V1-supplement-1.pdf]

bioRxiv preprint doi: https://doi.org/10.64898/2026.09.08.750063; this version posted September 14, 2026. The copyright holder for this preprint (which was not certified by peer review) is the author/funder. This article is a US Government work. It is not subject to copyright under 17 USC 105 and is also made available for use under a CC0 license.

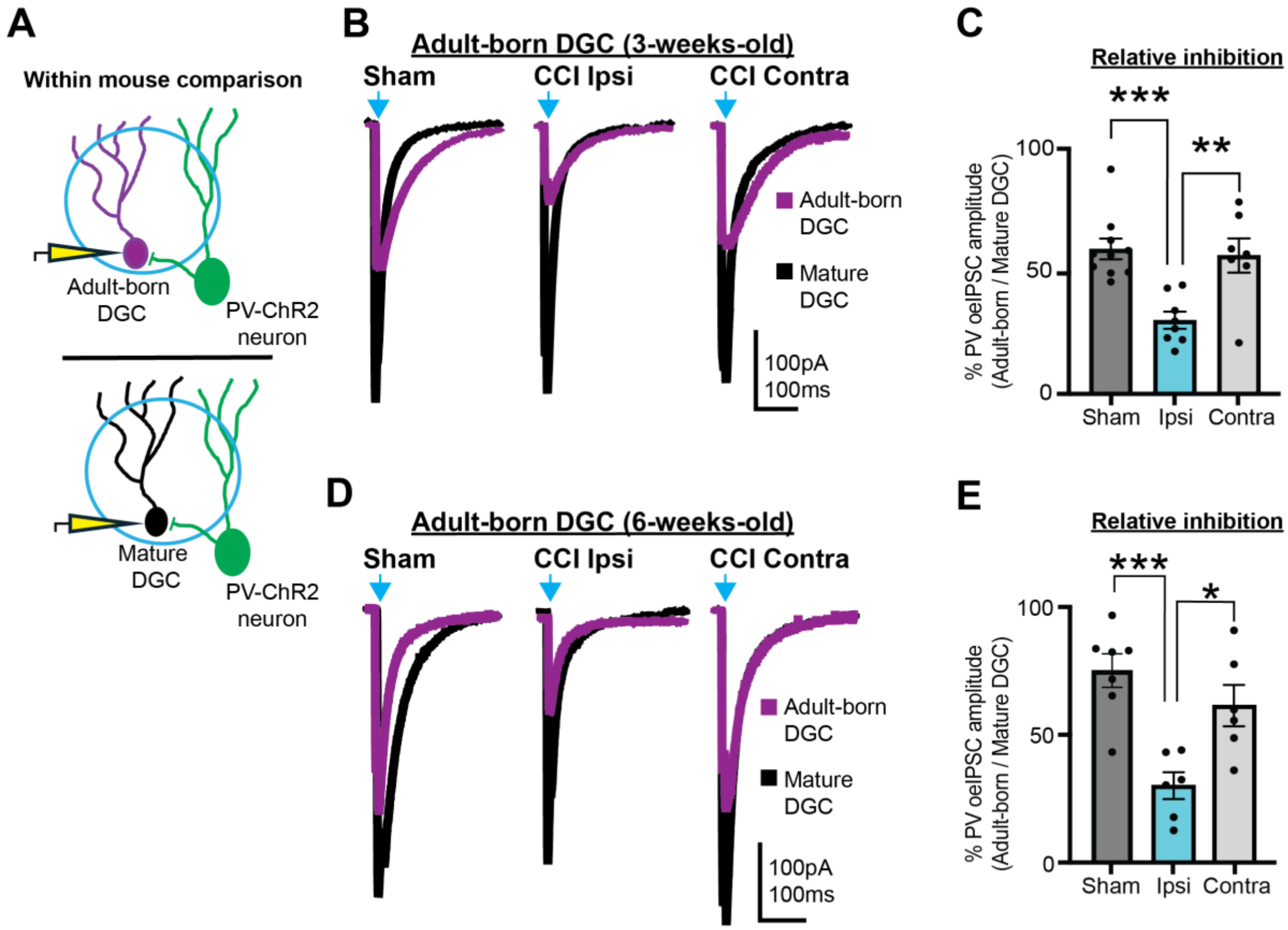

Supplemental Figure 1: Within-mouse comparisons demonstrate selectively reduced functional innervation of adult-born DGCs by PV cells after TBI. A) Schematic of within-animal comparisons of PV oeIPSCs in adult-born vs mature DGCs from the same animal/hemisphere. B) Representative PV oeIPSCs recorded from 3 week old adult-born (magenta) and mature (black) DGCs following Sham or CCI injury; blue arrow indicates time of light stimulation. Each pair of overlaid traces were recorded from the same animal/hemisphere. C) PV oeIPSC amplitudes in adult-born DGCs were consistently smaller than those recorded from their mature counterparts, but this ratio was further reduced in adult-born cells ipsilateral to CCI relative to Sham and CCI contralateral controls (Sham, n = 10 adult-born/ mature pairs, 6 mice; CCI Ipsi, n = 8 adult-born/ mature pairs, 4 mice; CCI Contra, n = 7 adult-born/ mature pairs, 4 mice; ** $p < 0.01$, *** $p < 0.001$). D) Representative PV oeIPSCs recorded from 6 week

bioRxiv preprint doi: https://doi.org/10.64898/2026.09.08.750063; this version posted September 14, 2026. The copyright holder for this preprint (which was not certified by peer review) is the author/funder. This article is a US Government work. It is not subject to copyright under 17 USC 105 and is also made available for use under a CC0 license.

old adult-born and mature DGCs following Sham or CCI injury; blue arrow indicates time of light stimulation. Each pair of overlaid traces were recorded from the same animal/hemisphere. E) Similar to PV oeIPSCs in 3 week old adult-born cells, relative PV-mediated inhibition of 6 week old DGCs is reduced ipsilateral to CCI relative to Sham and CCI contralateral controls (Sham n = 7 adult-born/ mature cells, 6 mice; CCI Ipsi n = 6 adult-born/ mature cells, 4 mice; CCI Contra n = 6 adult-born/ mature cells, 5 mice; ** $p < 0.01$, *** $p < 0.001$).

bioRxiv preprint doi: https://doi.org/10.64898/2026.09.08.750063; this version posted September 14, 2026. The copyright holder for this preprint (which was not certified by peer review) is the author/funder. This article is a US Government work. It is not subject to copyright under 17 USC 105 and is also made available for use under a CC0 license.

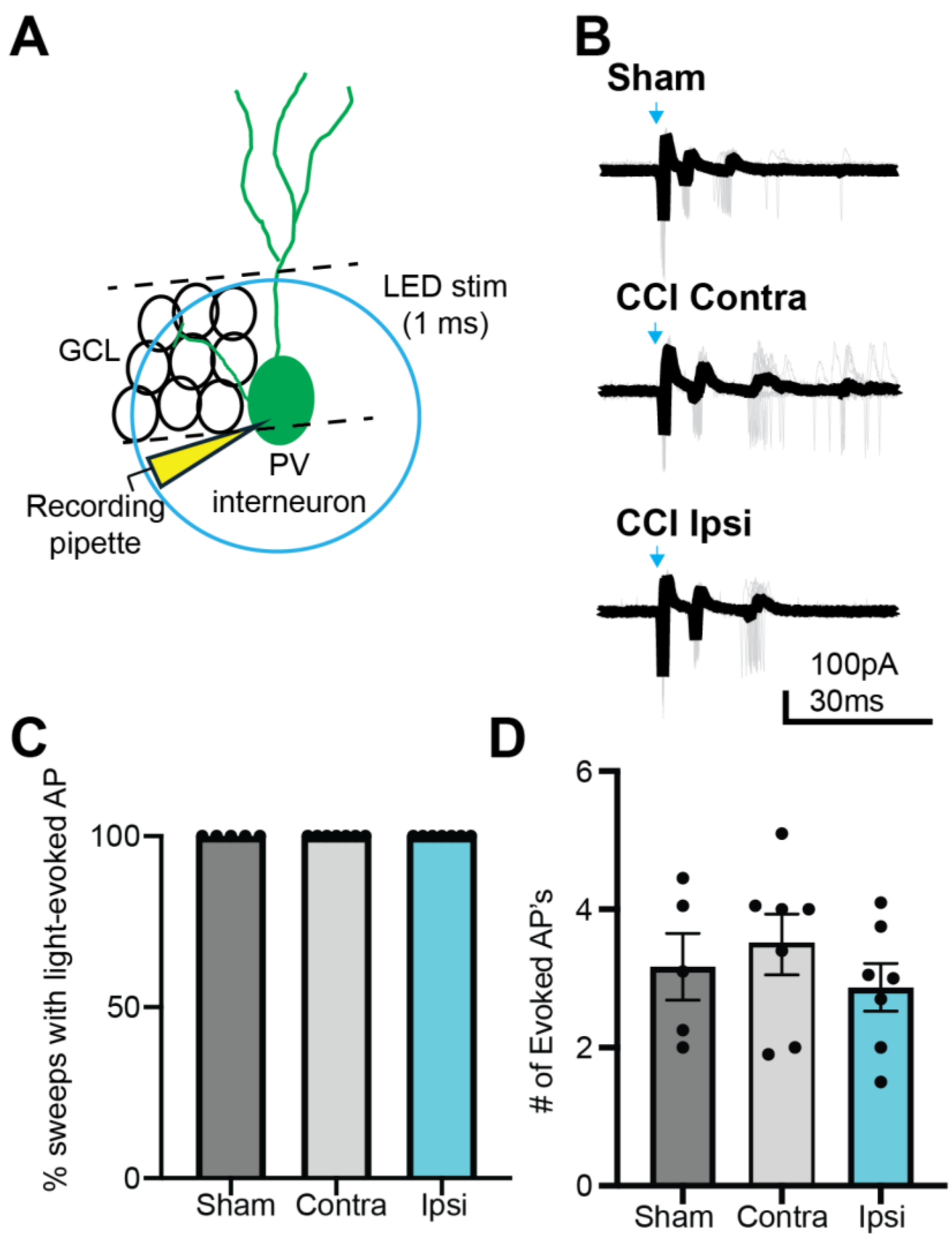

Supplemental Figure 2: Optogenetic activation of PV neurons is not altered by CCI. A) Recording schematic. Cell-attached recordings were obtained from ChR2-expressing parvalbumin (PV) neurons in the dentate gyrus during 1ms LED light (470nm) stimulation to evoke action potentials (APs). B) Representative optogenetically-evoked APs in PV neurons from Sham and CCI animals following LED stimulation; single sweeps shown in grey with averaged trace superimposed in black. C) ChR2-expressing PV neurons in the dentate gyrus fired at least one AP in response to light stimulation in 100% of sweeps (20 sweeps per neuron)

bioRxiv preprint doi: https://doi.org/10.64898/2026.09.08.750063; this version posted September 14, 2026. The copyright holder for this preprint (which was not certified by peer review) is the author/funder. This article is a US Government work. It is not subject to copyright under 17 USC 105 and is also made available for use under a CC0 license.

regardless of injury condition. D) The average number of light-evoked APs in PV-ChR2+ neurons was not different between experimental groups (n.s. for all groups).
